# Estimates of individual muscle power production in normal adult walking

**DOI:** 10.1186/s12984-017-0306-2

**Published:** 2017-09-11

**Authors:** Ross A. Bogey, Lee A. Barnes

**Affiliations:** 1 Physical Medicine and Rehabilitation Residency Program, Casa Colina Hospital and Centers for Healthcare, 255 East Bonita Avenue, Pomona, CA 91769 USA; 2 B&L Engineering, 1901 Carnegie Avenue, Suite Q Santa Ana, Santa Ana, CA 92705 USA

## Abstract

**Background:**

The purpose of this study was to determine the contribution of individual hip muscles to the net hip power in normal adult self-selected speed walking. A further goal was to examine each muscle’s role in propulsion or support of the body during that task.

**Methods:**

An EMG-to-force processing (EFP) model was developed which scaled muscle-tendon unit (MTU) force output to gait EMG. Active muscle power was defined as the product of MTU forces (derived from EFP) and that muscle’s contraction velocity. Passive hip power was estimated from passive moments associates with hip position (angle of flexion (extension)) and the hip’s angular velocity. Net hip EFP power was determined by summing individual active hip muscle power plus the net passive hip power at each percent gait cycle interval. Net hip power was also calculated for these study participants via inverse dynamics (kinetics plus kinematics, KIN). The inverse dynamics technique – well accepted in the biomechanics literature – was used as a “gold standard” for validation of this EFP model. Closeness of fit of the power curves of the two methods was used to validate the model.

**Results:**

The correlation between the EFP and KIN methods was sufficiently close, suggesting validation of the model’s ability to provide reasonable estimates of power produced by individual hip muscles. Key findings were that (1) most muscles undergo a stretch-shorten cycle of muscle contraction, (2) greatest power was produced by the hip abductors, and (3) the hip adductors contribute to either hip adduction or hip extension (but not both).

**Conclusions:**

The EMG-to-force processing approach provides reasonable estimates of individual hip muscle forces in self-selected speed walking in neurologically-intact adults.

## Background

The role of individual hip muscles in normal walking has not been fully described. A more complete understanding of each muscle’s role could be established by knowledge of that muscle’s force output and power generation. However, direct measurements of muscle force has been obtained for only a few lower extremity muscles (i.E. *Achilles* tendon [1, 2]), and the techniques used to directly record muscle forces – even when possible – are not practical in a clinical environment. Muscle power is related to muscle force, in that it is the product of force output and muscle-tendon unit (MTU) contraction velocity. Joint power – that is, the power produced by synergistic muscles – may be used to establish the capacity of muscle groups to generate or restrain movement [3–12]. Concentric power typically produces motion, while negative power implies motion restraint. A possible confound is that the presence of muscle power has been used as a proxy for muscle activity, yet studies of amputee locomotion [13] demonstrate non-zero power at the prosthetic ankle. Further, isometric contractions have zero power, due to a contraction velocity of zero. Nonetheless, the power produced by any muscle cannot be directly determined. Hence other methods are required.

An impediment to easy determination of muscle power – and the potential role of individual hip muscles – is that the hip has more muscles than are necessary to perform basic movements. This overabundance of muscles leads to statistical indeterminacy (more unknown muscle forces than solution set of equations). Power calculation approaches thus are based on inverse dynamics techniques. This method solves the actuator-redundancy problem. However, conventional power analysis cannot define the unique power contribution of a single muscle, except in uncommon cases where only a single muscle is responsible for the observed movement. Co-contraction of agonists and antagonists is a further confound. As a result there may not be a direct link between a muscle’s power output (via inverse dynamics techniques) and that muscle’s true role in gait. These factors show that the muscle power estimates from inverse dynamics can include muscle force, joint angular velocities and other, undefined variables.

Neuromuscular modeling techniques have produced reasonable estimates of in vivo ankle muscle power [14]. It was the purpose of this study to examine if those techniques could be applied to at the more proximal hip joint to determine individual hip muscle power production in normal, self-selected-speed walking.

## Methods (overview)

Hip power values at self-selected speed walking were determined by (*i*) inverse dynamics (kinematics plus kinetics, KIN) and (*ii*) an EMG-to-force processing (EFP) model during the same gait trial for each study participant. Net power for each method was statistically compared at 1 % gait cycle intervals. Closeness of fit between the EFP and KIN power curves was used to validate the EFP model. If the resulting power curves were sufficiently similar, the EFP power estimates were deconstructed to analyze the power contribution and role of individual hip muscles in normal gait.

### Subjects

Twenty (20) adult males with no history of neuromusculoskeletal disease were recruited to participate in the study. Two subjects did not participate in repeat gait trials, and their data were not further examined. The eighteen remaining study participants had a mean age of 29 ± 3.1 years (range 24–34 years), and a mean mass of 76.0 kg (± 6.75 kg). Due to the comprehensive number of muscles examined each study participant performed two self-selected speed walking trials, with data selected from each trial. In normal adults the examined gait parameters are highly consistent across trials [15], and the use of multiple sessions did not confound the results. The mean gait velocity for these subjects was 81.9 ± 3.7 m per minute. Subjects consented to participate following explanation of the procedure and review of the informed consent, as approved by the Institute Review Board, and signed the Rights of Human Subjects form.

### Kinematics and kinetics (KIN)

Gait data acquisition and processing steps used in this study have been described in greater detail elsewhere [15]. The primary focus of this analysis was the power generated at the hip through the entire gait cycle. Kinematic, kinetic, footswitch and EMG data were simultaneous acquired while study participants walked at their preferred, self-selected speed. Study participants began each trial standing at the end of the 12 m level walkway. After a few strides they reached their self-selected walking speed, which they maintained across the middle 6 m of the walkway. A few strides were needed to decelerate at the end of the walkway. As our goal was to examine “typical” (i.e. constant velocity) gait the data analysis was thus limited to the portion of the gait trial where study participants were within the middle 6 m of the walkway. Study participants wore appropriate clothing, consisting of a light shirt, shorts and their own flat-soled shoes. Contact-closing footswitches (B&L Engineering, Tustin, CA) were placed in the participant’s shoes during the trials to determine stance and swing times. Twenty round reflective markers were used to determine joint centers of rotation, with markers placed over anatomic landmarks as described by Kadaba [16].

Study participants performed several preliminary trails to become acclimated to the test environment. As normal adult gait is essentially symmetric data analysis was restricted to the right leg, only. Data were averaged across five typical trials for each study participant.

Motion data was sampled at 100 Hz with an eight camera system (Model T20, Vicon Motion Systems, Oxford, England). Marker coordinates were bi-directionally smoothed with a fourth-order Butterworth filter with an effective cutoff frequency of 6 Hz. Linear velocities and accelerations, and angular position, velocity, and acceleration of segments were determined as described elsewhere [10]. Ground reaction force (GRF) data was collected at 600 Hz with paired force platforms (Type OR6–5, Advanced Medical Technology Incorporated, Newton, MA). GRF data was smoothed, the center of pressure location was determined [10], and motion and ground reaction force data were temporally matched. Mass and center of mass locations were determined [17]. Three-dimensional joint moments and power were computed at the hip via Newtonian mechanics using in-house software. Mechanical power at the hip was based on the kinetics and kinematics of the individual body segments (allowing no transfer of energy between segments) and was defined as dot product of the hip moment and angular velocity vectors

$$ {P}^{KIN}={M}^{KIN}\cdot {\omega}^{KIN}. $$

Joint position data were also used to determine muscle lengths via a force processor model (*below*).

### EMG-to-force processing — Force processor

The neuromusculoskeletal model included lower extremity skeletal structures plus fourteen muscles crossing the hip. Full details of the SIMM-based Force Processor model are presented elsewhere [18]. The model consisted of three-dimensional representations of the bones and muscle-tendon paths, kinematic descriptions of the ankle, knee and hip, and a nominal biomechanical model of each musculotendinous unit (MTU).

The isometric force generating properties of each of the fourteen muscles were derived by scaling a Hill-based model [19]. The scaled model was matched to each study participant’s unique limb segment-mass and segment-length characteristics. The effect of muscle force-velocity relations, muscle contraction history and contraction type were considered [20].

### Methods: EMG-to-force processing — Dynamic electromyography (EMG)

Electromyographic (EMG) was obtained from the following hip muscles: Gluteus Medius, Gluteus Minimus, Tensor Fascia Lata, Adductor Longus, Adductor Brevis, Gracilis, Rectus Femoris, Iliopsoas (combined Iliacus and Psoas), Sartorius, Semitendinosus, Semimembranosus, Biceps Femoris (long head), Adductor Longus, and Gluteus Maximus. Electromyographic activity was recorded with bipolar 50 μ stainless steel wire electrodes insulated with polyimide except for 2 mm exposed tips. The electrodes were inserted into the tested muscles with a 20-gauge hypodermic needle [21] at insertion sites for each muscle as described by Delagi [22]. A single ground electrode was placed over a bony landmark on the lower extremity (e.g. greater trochanter). Electrode placement was confirmed by electrical stimulation of the muscle via the indwelling electrode and by voluntary muscle contraction.

The EMG system bandwidth was 40-1000 Hz with an overall gain of 1000 [23] (Model MA-300, Motion Laboratory Systems, Baton Rouge, LA). EMG was normalized to a maximum voluntary isometric contraction [24]. Each manual muscle test was performed [25] while electromyographic activity (in mV) was recorded. One hundred percent maximum voluntary contraction (100%MVC) was the one-second interval with the highest mean intensity during a five-second manual muscle test. A second maximum muscle test was performed at the end of the gait trials to insure continued integrity of the electrode insertion.

The gait cycle interval and foot support patterns were recorded with footswitches. EMG and footswitch data were collected at 2500 Hz. The digitized EMG data were rectified and integrated. A resting run was used to correct for baseline noise. Stride data were divided into 1%GC intervals with %MVC determined at each %GC interval.

Several sequential processing steps were necessary to determine the EMG input to the force processor. First, linear envelopes (LE) were generated from the rectified, integrated EMG signal for each stride (Olney, 1985). Next, within-subject electromyographic profiles for each muscle were then established from individual stride EMG [26]. The final step in EMG processing involved accounting for the temporal lag between within-subject electrical activity (EMG) and force production (electromechanical delay, EMD [27–29]). A second-order critically-damped Butterworth low-pass digital filter was used to match the EMG input to force output [30, 31]. The post-processed EMG profile ε(t) was defined by

$$ \varepsilon (t)=\gamma \left({\rho}_1E(t)+{\rho}_2E\left(t-1\right)+{\rho}_3E\left(t-2\right)+{\rho}_4\varepsilon \left(t-1\right)+{\rho}_5\varepsilon \left(t-2\right)\right) $$

where γ was ratio E_max_/ε_max_, *E* the %MVC for the within-subject EMG profiles, ε the %MVC for the within-subject EMG profiles following filtering, and the values of the constants *ρ*_1_ − *ρ*_5_ were derived from the sampling frequency (F_s_) to cutoff frequency (F_c_) ratio (F_s_/F_c_). A single, empirically-established F_s_/F_c_ ratio was used for all muscles and was similar to previous reported values [14, 20, 32, 33]. The post-processed EMG profiles were the neural input (*activation dynamics*) to the force processor model. The use of the constant (γ) lessened further attenuation of the EMG signal as a result of signal processing.

Muscle-tendon unit force (F^MTU^) and muscle lengths (L^MTU^) were determined at each data acquisition interval. The contraction velocity for each muscle (v^MTU^) was obtained by mathematical differentiation of MTU length. Concentric contractions were defined as positive. Power produced by each muscle was the dot product of that muscle’s force and co-linear contraction velocity vectors

$$ {P}_{active}^{MTU}={\boldsymbol{F}}_{\boldsymbol{active}}^{\boldsymbol{MTU}}\cdot {v}_{\boldsymbol{active}}^{\boldsymbol{MTU}}. $$

### Passive hip muscle power

Previous work [34] demonstrated a substantial contribution to the net hip moment by passive tissues crossing the joint. Direct measurement of passive hip power was not possible. Hence representative estimates of the hip passive power were derived from values for the hip moment – based on joint position – from the literature and the temporally-associated hip angular velocity. Similar to Bogey et al. [34] an a priori decision to use a liberal estimate of the passive moment was made. Thus the passive power contribution was the product of the (estimated) passive moment and the hip’s angular velocity

$$ {P}_{passive}^{EFP}={M}_{passive}^{EFP}\cdot {\omega}^{hip}. $$

The net EFP power was defined as the sum at each percent gait cycle of the active and passive power

$$ {P}_{net}^{EFP}={P}_{active}^{EFP}+{P}_{passive}^{EFP} $$

where the EFP net hip power $\left({P}_{net}^{EFP}\right)$ for each study participant was obtained by summing the power produced by all fourteen hip muscles at each %GC interval. KIN net hip moments were determined via standard inverse dynamics techniques (above) and the across-subject mean EFP hip power was statistically compared with the mean KIN hip power determined for these same individuals

$$ {P}^{KIN}\approx {P}_{net}^{EFP}. $$

Inverse dynamics (kinetics plus kinematics, KIN) hip power was determined for each study participant, and the mean magnitude for each were compared at each %GC interval

$$ {\sum}_{i=1}^N{P}^{EFP}\approx {\sum}_{i=1}^N{P}^{KIN}. $$

### Statistics

The EFP hip power was determined at each percent gait cycle interval then combined across study participants. Mean EFP hip power was compared with mean KIN hip power, also at %GC intervals. Inspection of our pilot data indicated that the model and experimental data satisfy parametric statistical assumptions [35]. Thus the correlation between EFP and KIN hip power was calculated, and consistent with other EMG-to-force processing models an *r* > 0.70 was required for Model acceptance. This number shows a high level of equivalence between experimental and Model values, and been used at the benchmark for validation of other muscle models [20].

## Results

**Fig. 1:**
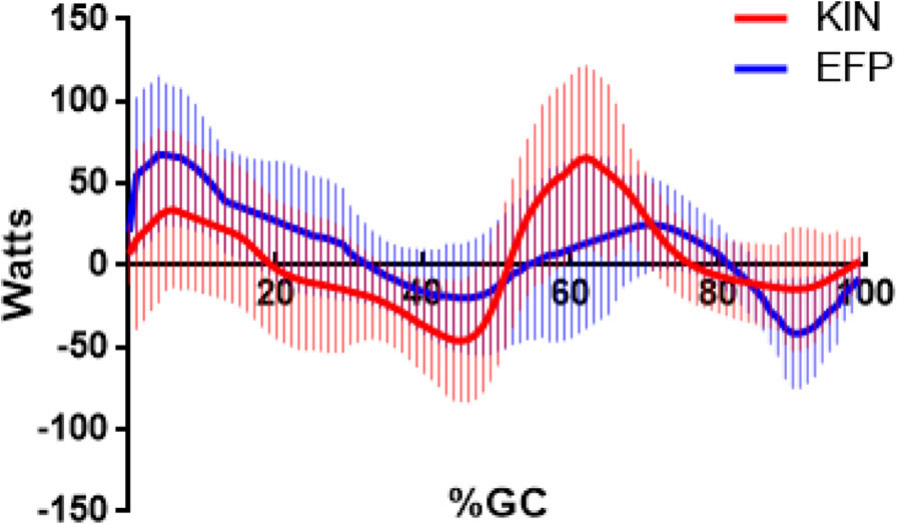
Mean +/- one standard deviation KIN (Blue line) and EFP (Red line) sagittal plane hip powers are shown. Percent gait cycle is shown on the abscissa. End of stance phase occurred at ≈ 62%GC. Concentric contractions are positive

EFP and KIN hip power were highly correlated and similar in magnitude (*r* = 0.74, Fig. 1). Semimembranosus produced the greatest amount of positive power (33 W, which occurred in early stance (6%GC)). Semimembranosus and Biceps Femoris produced 10 W and 11 W of power, respectively, during stance. The largest single muscle hip power magnitude was also produced by Semimembranosus in swing (−55 W). Biceps Femoris also had large negative power (−24 W), while the swing phase peak power produced by Semitendinosus was modest (−5 W).

The KIN and EFP curves were similar in shape; that is, they tended to increase (decrease) in value at corresponding times (Fig. 1). However, there were substantial differences in hip power at three intervals in the gait cycle. KIN predicted higher negative power in late single support (≈ − 40 W). This value was surprising, as there is little hip muscle activity (as noted by the paucity of dynamic EMG) during this epoch. In a similar manner pre-swing and early swing phases were notable for elevated KIN positive power, while EFP values remain low. This is little doubt that the hip flexes vigorously in this interval (hence high KIN power), while the only hip flexor producing force at that time was the Rectus Femoris. Finally, EFP indicates much higher negative power than KIN in late swing. This was coincident with high force output by all five hip extensors with active lengthening by all hamstrings and Gluteus Maximus (Adductor Magnus activity is described below).

Notable power production by other muscles included the Gluteus Medius (17 W, in single support). The fact that positive power by this muscle was greater in magnitude than its peak negative power was unforeseen, as its role in stance is generally described as limiting contralateral lateral tilt of the torso (negative power). The Rectus Femoris (RF) produced -14 W during the transition from stance to swing phases. The hip had begun flexing before the onset of RF force and power, while concurrent knee flexion lengthens the bi-articular RF. Thus there was a stretch-shorten cycle for this muscle, with expected augmentation of muscle force during the concentric contraction phase [36, 37]. In contrast the uni-articular Iliopsoas was limited to concentric work to enhance hip flexion (peak power = 7 W). Early swing phase hip flexion was assisted by modest positive power produced by the Gracilis and Sartorius (<5 W, each).

## Discussion

### Model validation

One goal of this research was to examine if an approach that applied a study participant’s EMG input and anthropometry to a generic neuromuscular model would yield reasonable estimates of in vivo muscle power. A comparison of directly-measured muscle power with the EMG-to-force processing model estimates would be – in theory – the best way to validate this model. However, direct measurement of in vivo muscle power at the human hip is not possible. Thus inverse dynamics techniques are often used as a proxy (“gold standard”). The fact that the net EFP (passive plus active) and KIN hip power curves both were closely correlated *and* favorably similar in magnitude supports the use of EMG-to-force processing to obtain reasonable estimates of in vivo hip muscle power.

### Modeling of individual muscles

Fourteen muscles that cross the hip were included in the model. Fundamentally the muscles were defined by all muscle fibers bounded by the sarcolemma and the associated proximal and distal tendon structures. Further partitioning of the tested hip muscles has been suggested in the literature. The Adductor Magnus is reported to have four distinct functional segments [38]. It may be clinically subdivided into an adductor portion and a portion that is functionally associated with hip extension [39]. This unique muscle is also dually-innervated, which makes the decision to model it as one-, two- or four muscles even more complex. During preliminary trials dynamic EMG was acquired with intramuscular electrodes positioned in several different sites within the muscle. Its EMG profile was out-of-phase with other hip adductors. For consistency a posteriorly-located site (which also had the greatest gait voltage output) was used to obtain Adductor Magnus EMG. Inclusion of the anterior aspects of the muscle augmented the muscle’s physiological cross-sectional area, and the resulting power estimates may be slightly overestimated. Further, Gluteus Medius may have three distinct anatomical segments [40] (posterior, lateral and anterior) while the Gluteus Minimus has distinct fiber orientation in its anterior (vertically-oriented fibers) and posterior (horizontally-oriented fibers) aspects [41]. There is no substantial evidence of marked differences in gait EMG for either of the aforementioned hip abductors, thus division of these muscles into smaller functional units was briefly considered yet ultimately rejected. In contrast, two muscles that are sometimes considered to have similar functions were combined. Iliacus and Psoas (“Iliopsoas”) were modeled as a single entity with shared distal insertion and their combined physiological cross-sectional areas. Subsequent EMG-to-force processing models might examine the respective contribution of these “muscles within muscles”, with a goal of improving post-surgical outcomes and to better target rehabilitation programs.

While the vast majority (and largest) of the hip muscles were included in this model, a few muscles were not tested (Inferior and Superior Gemellus, Obturator, Piriformis). These small, deep muscles are rarely examined in clinical gait studies, and due force and power production are relatively trivial. Thus we anticipated little error in the net EFP power as a consequence of not including these muscles in the model.

### Potential error sources

Unfortunately there is no perfect neuromuscular model. Like all models the EFP approach examined has certain built-in “error sources”. For this model the gait muscle forces (and by amplification, muscle power contribution) required accurate maximum muscle tests (MMT) to scale MTU forces. Any imprecision in obtaining an accurate MMT could lead to errors in force estimates, which could – in turn – then result in errors in power assessment. The risk here is that the MMT may not represent true maximum effort. This procedural error would result in an overestimate of MTU forces (and ultimately muscle power). All efforts were made to minimize this risk, including careful positioning of the limb during testing [25], plus obtaining more than one test (pre- and post-gait trial) to determine the best reference value. These steps diminish (but don’t eliminate) the possibility of less-than-maximal effort during muscle testing. Yet an examination of the respective power curves (Fig. 1) confirms that errors in muscle forces were minor.

While EMG acquisition with wire electrodes can be technically challenging, there use was necessary to record activity of deeply-positioned muscles and to minimize intramuscular crosstalk. It is customary to use surface electrodes for EMG data acquisition in gait analysis. Intramuscular recordings are less common. In part, this is due to the need for highly-trained individuals for signal acquisition when using wire (intramuscular) electrodes, and to a lesser extent mild discomfort on the part of study participants. A concern has been extrapolating the results from the smaller muscle volume that is recorded with wire electrodes to the entire muscle. This is an important question, particularly when attempting to make reasonable estimates of muscle force and power. However, the reservations related to generalizing the wire EMG data to the entire muscle – at least in neurologically-intact individuals – are unfounded. Previous work has shown that when the wire EMG signal has been normalized to a reference standard (in this case, a maximum muscle test) there were no significant differences in the wire and surface EMG signal timing or magnitude [15]. Although surface electrodes could have been used to record data from superficial muscles they could not have been used to record EMG from many of the deep muscles examined here. Thus a single electrode type – wire electrodes – was used to examine all tested hip muscles to remove a potential confound.

### Passive hip power

There is uncertainty in the precision of hip passive power estimates, as these values cannot be obtained directly. Further, previous work that estimated passive hip moments (a contributor to passive hip power) only examined the sagittal plane component of passive (non-contractile) tissues. Clearly these same tissues also restrain (and possibly generate) motion in the frontal and transverse planes, yet even passive moments in the aforementioned planes are not available. However, direct measurement of passive power is at odds with one goal of this research – that is, to get reasonable estimates of individual muscle power without (impractical) direct measurements. The passive power contribution influences the decision to accept or reject the model; however, it has no direct impact on the calculation of power produced by any tested muscle. Thus this potential confound was limited to epochs where both active and passive were present. Passive power was present in only 1/3 of the gait cycle (34–68%GC [34]).

### Role of individual muscles

As a function of their larger physiological cross-sectional area the muscles crossing the hip have more force-generating potential than either knee or ankle muscles. Yet ankle forces [20] and power [14] were substantially higher than hip forces [34] and power in normal adult gait. The peak power magnitude shown here (55 W) was slightly less than 50 % of Soleus peak gait power (110 W [14]). This highlights that while hip muscle strength and coordination are extremely important in the performance of many movements (e.g. running, stair climbing) they may be relatively less important in performing self-selected speed level walking.

One important finding was the near-ubiquitous occurrence of stretch-shorten type contraction by the hip muscles. Ten of the fourteen hip muscles were actively eccentrically, then immediately concentrically contracted during activation. Here energy was stored in actin-myosin cross-bridges, and within the tendons that are in-series with the muscle fibers. This muscle contraction sequence is associated with enhanced force output at negligible metabolic cost [37, 42–46].

### Hip abductors

**Fig. 2:**
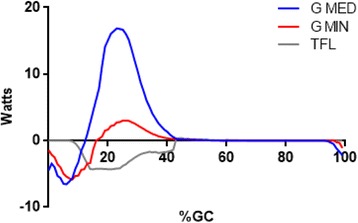
Mean EFP power for the hip abductors are shown. Gluteus Medius (Blue line), Gluteus Minimus (Red line), and Tensor Facia Lata (Gray line) power are presented. Concentric contractions are positive

This approach gave insights into the roles of several muscles that have not been previously described. Mechanical stability in the frontal plane is dependent on appropriate power production by the hip abductors [47, 48]. Early stance negative hip abductor power (Gluteus Medius, Gluteus Minimus) lessens the medial-lateral instability (Fig. 2). While a virtual ocean of literature has been devoted to the role of the hip abductors in preventing excessive contralateral lateral tilt in early stance [39, 49, 50], the fact that these muscles concentrically contract (positive power) to restore a vertical torso posture (and keep the eyes relatively level) has received only a trickle of prose. A surprising finding was the relatively modest negative and position power produced by the hip abductors. Previous work has shown them to have the greatest peak force output for hip muscles [34] yet power production was exceeded by several hip extensors. While their force production was high, muscle contraction velocity was low. Thus the power produced to restrain (in weight acceptance) or generate motion (in single support) is unexceptional. Positive power was limited to the gluteal abductors, as TFL produced only negative power. This finding likely reflects the fact that the TFL was also directly involved in knee joint kinematics.

### Hip adductors

**Fig. 3:**
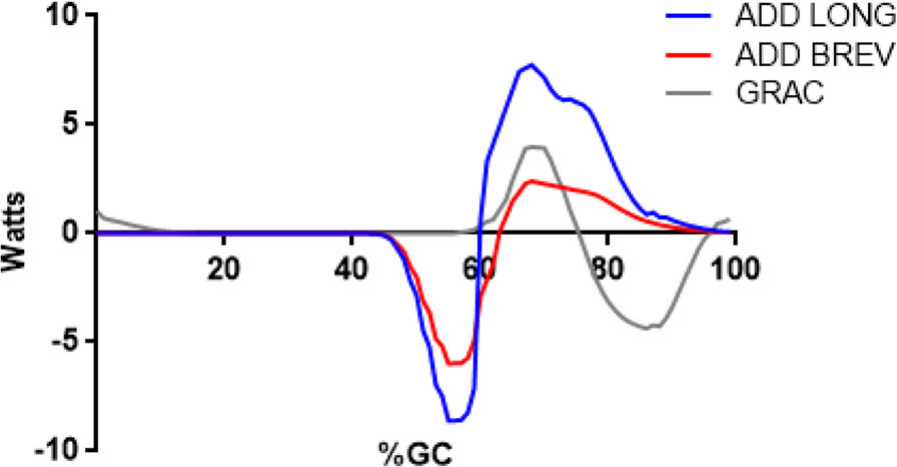
Mean EFP power for the hip adductors are shown. Adductor Longus (Blue line), Adductor Brevis (Red line), and Gracilis (Gray line) power are presented. Concentric contractions are positive

Initial hip adductor power was negative. Both Adductor Longus and Adductor Brevis were eccentrically contracted (negative power) in stance to attenuate hip abduction, later shortened (concentric contraction, positive power) as swing begins (Fig. 3)**.** The onset of Gracilis force and power temporally matched positive power produced by Adductor Longus and Adductor Brevis, albeit without a preparatory lengthening contraction. Positive adductor power peaked shortly after swing phase initiation.

Several researchers have noted that the hip adductors (Adductor Longus and Brevis) bring the swing leg toward the mid-line during swing. The muscle work (positive power) would narrow the base of support during gait, and decrease the metabolic work demands of walking. Yet negative power produced by these muscles in pre-swing was higher magnitude than the positive swing phase power generated. Adductor negative power began as their antagonist group (hip abductors) terminated force (≈45%GC). Thus the first task for the hip adductors may be to terminate stance phase hip abduction, then after being stretched, perform their more-frequently described function to bring the swing phase limb toward the midline in swing.

### Hip flexors

**Fig. 4:**
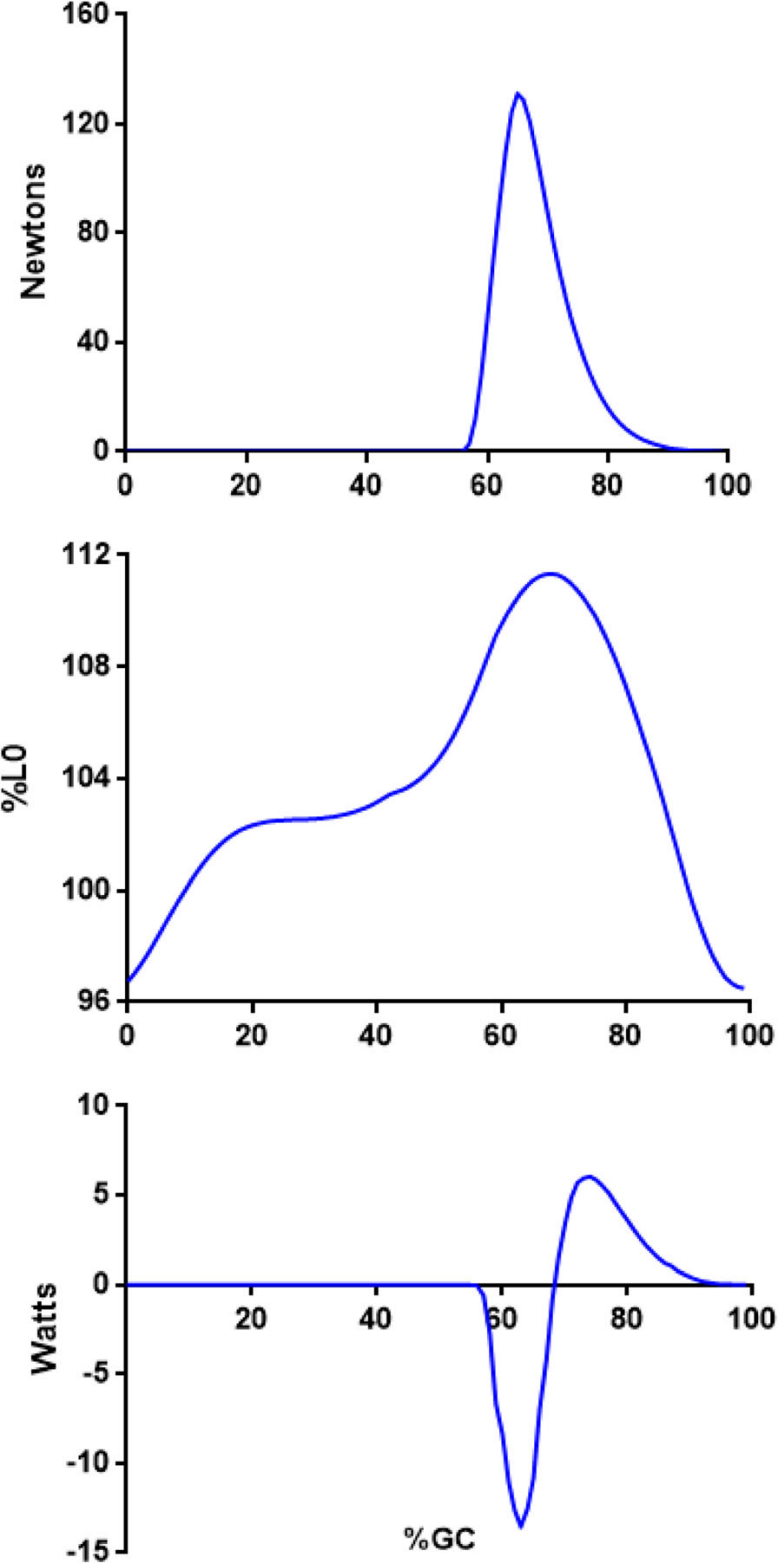
Mean Rectus Femoris gait forces (top), relative muscle length (middle), and power are shown. Muscle length is expressed as percent anatomic length (hip and knee at anatomic position). Positive power (bottom) correlates with concentric muscle contractions

**Fig. 5:**
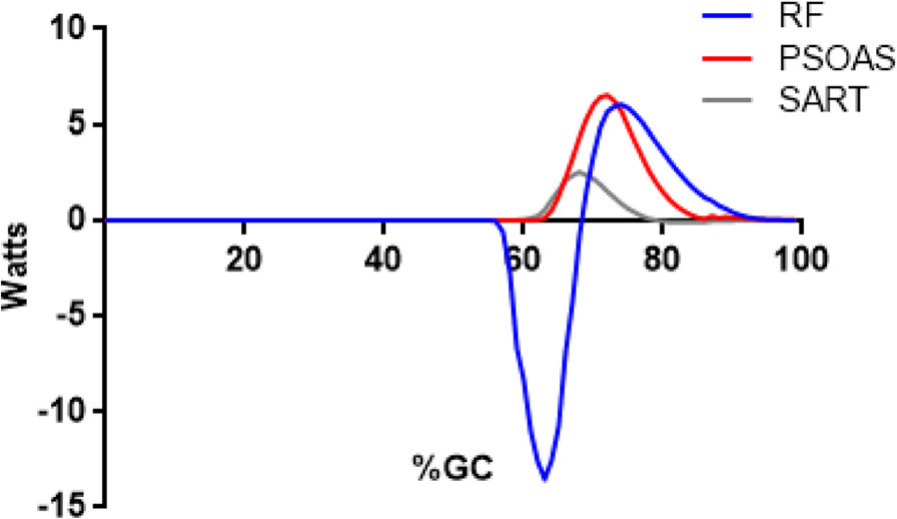
Mean EFP power for the hip flexors are shown. Rectus Femoris (Blue line), Iliopsoas (Red line), and Sartorius (Gray line) power are presented. Concentric contractions are positive

The transition from stance-to-swing was significant for negative power produced by only a single hip flexor (Rectus Femoris, RF). This bi-articular muscle can produce force and power at the hip and/or knee (Fig. 4). As noted, one limitation of the EFP methodology is that muscle power cannot be partitioned to adjacent joints where a bi-articular muscle is producing force (and power). The hip was flexing as RF force production began, yet the knee was also flexing. The observed knee and hip motion may be in response to rapid ankle plantar flexion and its effect on the more proximal joints (as part of a linked triple-pendulum configuration). As the rate of knee flexion was greater than hip flexion (Fig. 4, top), RF was extended beyond its anatomic length (L_0_), and net negative RF power was generated. The other hip flexors (Iliopsoas, Sartorius) began force production that nearly coincided with the beginning of swing, and produced only positive power (Fig. 5)**.** Rectus Femoris had the highest hip flexor force output, yet Iliopsoas generated slightly more positive power (greater concentric contraction velocity) to advance the trailing limb. By mid-swing hip flexion was passive. That is, hip flexor power ended in mid-swing, and yet hip flexion continued (passively) until slowed in late swing via concentric work by the hip extensors (*above*).

### Hip extensors

**Fig. 6:**
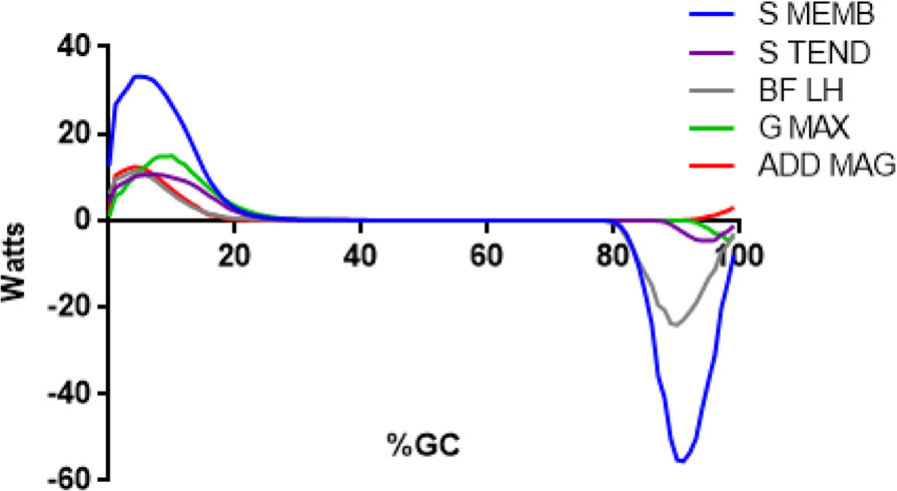
Mean EFP power for the hip extensors are shown. Semimembranosis (Blue line), Semitendinosis (Purple line), Long Head of the Biceps Femoris (Gray line), Gluteus Maximus (Green line), and Adductor Magnus (Red line) power are presented. Concentric contractions are positive

As previously noted, one of the shortcomings of the EFP approach is the inability to determine how power might be shared at adjacent joints by a bi-articular muscle. Five muscles can extend the hip, yet of these only the three hamstring muscles (Semimembranosus, Semitendinosus, long head of Biceps Femoris) are recruited in mid-swing (Fig. 6). That is, they slow mid- and terminal-swing knee flexion by producing negative power at that joint. As swing advanced (from mid-swing to terminal swing) the hamstrings’ combined negative power is enhanced by Gluteus Maximus force and power production. The role of the remaining hip extensor – Adductor Magnus – is more complex. During late swing Adductor Magnus simultaneously produces positive power to adduct the limb and negative power to assist in slowing hip flexion. Its MTU length did not substantially change, minimal power was generated, and the net power that was produced is positive. As stance phase began all hip extensors produced positive power. The presence of (exclusively) positive power by the hip extensors in stance is at odds with the belief that a key role of these muscles was to prevent “jack-knifing” [39] at the hip during the transition from swing-to-stance. The data presented suggests that their collective responsibility is to generate motion to horizontally propel the body’s center of mass over the fixed foot. Positive hip extensor power continues into single support. With the cessation of hip power continued hip extension is passive.

The relative timing of the hip adductor muscles warrants further comment. For Adductor Longus and Adductor Brevis both force [34] and power production diminish then terminate in late swing (Fig. 3). Yet the largest hip adductor – Adductor Magnus – produced power that was essentially out-of-phase with the other primary hip adductors (Fig. 6). This finding may have important clinical implications. Focal tone reduction of Adductor Magnus associated with spastic gait (i.e. scissoring associated with Cerebral Palsy) may not treat the source of the problem and may simultaneously attenuate desired hip extension. Quantitative assessment (gait analysis) is suggested to minimize the risk of sup-optimal post-treatment outcomes.

## Conclusions

One aim of this study was to determine if an EFP approach could yield reasonable estimates of individual muscle power. This appears to be so. Kinetic analysis (KIN) served as the “gold standard” for EFP validation, and the EFP and KIN power curves were very similar (Fig. 1). While it is possible that there are self-cancelling errors, the closeness of fit between the EFP and KIN power curves indicates that the model’s power estimates are reasonable. It is feasible that the match of actual versus predicted power is closer for some muscles than for others. When compared to KIN methods one important distinction in the EFP approach is that the contribution of individual muscles can be determined. It is important to note that all input variables to the model (%MVC, EMD) were determined a priori, and that this approach was not a curve-fitting exercise.

The results presented are encouraging. When combined with individual muscle forces our understanding of normal gait can be substantially enhanced. There may be important clinical implications to this work, as rehabilitation devices and protocols are based – in part – on suppositions of which muscles and joints are essential to generate or restrain movement.

Thus far EMG-to-force processing models – including the one presented here – have been limited to estimating in vivo forces and (rarely) power in neurologically-intact individuals [20, 51–53]. Future studies are needed that successfully adapt the principles and techniques described to persons with neurologically disease or injury.

## Abbreviations

EFP: EMG-to-force processing
EMD: Electromechanical delay
EMG: electromyogram
GC: gait cycle
GRF: ground reaction force
Hz: Hertz
Kg: kilograms
KIN: combination of kinetics and kinematics
LE: linear envelope
M: Moment
MMT: Maximum muscle test
MTU: Muscle-tendon unit
MVC: maximum voluntary contraction
P: Power
RF: Rectus Femoris
TFL: Tensor Fascia Lata
W: Watts
ω: angular velocity
